# Outcomes of an indian intensive care unit in a tertiary hospital - a prospective observational study

**DOI:** 10.1186/2197-425X-3-S1-A760

**Published:** 2015-10-01

**Authors:** S Jakkinaboina, P Peddapyata, S Paul, R Celerappa, M Aleem

**Affiliations:** Apollo Health City, Critical Care Medicine, Hyderabad, India

## Introduction

The mortality and morbidity of the patients admitting to the intensive care unit is high all over world. The evidence based treatment can improve the outcomes in the intensive care unit.

## Objectives

The objectives of this study were to evaluate the effect of an in-hospital mortality and morbidity for the patients admitting to the intensive care unit in a tertiary care hospital with adherence to evidence-based guidelines.

## Methods

All the patients admitted to the ICU are enrolled except post cardiac arrest and paediatric patients. A prospective study was done for a period of 6 months from Feb 2014 to Aug 2014. The data was collected from patient records after ethics committee approval. The primary end point was in-hospital mortality. The secondary outcomes were hospital and ICU length of stay.

## Results

The total number of patients enrolled are 486.

Table 1 shows the data of the overall patients in the group.

**Table 1:** Data of the overall patients admitted to the ICU.

Variable	Mean n	standard deviation, (%)
Age in years	55.42	17.5
ICU length of stay in days	3.52	3.2
Hospital length of stay in days	9.11	9.24
APACHE II Score	14.72	8.2
SOFA Score	8.33	4.2
Male sex	282	(58.02)
Medical patients	386	(79.42)
Vasopressors	152	(31.28)
Mortality	36	(7.41)

The in-hospital mortality is 7.41%. The most common admission diagnosis is shock. Most common comorbidities are Diabetes and hypertension (38.68%) followed by diabetes(10.7%). Neutropenia is present in 2.88% patients. 80.25% patients received antibiotics before admission to ICU, Most common antibiotics used are BL+BLI (57.61%) combination followed by carbapenems(14.82%). Most common organ failure is lung (23.87%) followed by Renal failure(23.46%). RRT given for 22.22% patients. Tracheostomy was done in 4.53% patients. The factors which increased the mortality are increased ventilator days, increased APACHE II and SOFA score, shock as the diagnosis, comorbidities both diabetes and hypertension, presence of neutropenia, reintubation, sepsis, antibiotics before admission to hospital, lung and renal as organ failures and use of renal repalcement therapy. Almost the same factors increased the ICU and hospital length of stay. The ICU acquired infection total are 20patients (4.12%) has 65% ESBL(E. Coli, kleibsiella), MDR 25% (Kleibsiella, psedomonas) MRSA 5%, MSSA 2%, Fungal infections 3%. The hospital acquired infection (other wards) 12 patients(2.47%) with ESBL 70% and 30% are MDR organisms. The community acquired infections are 30% non ESBL, 40% ESBL, MDR 10% and remaining 20%. The data of survivors and non survivors are in table 2.

**Table 2:** The variables between survivors and nonsurviors.

Variable	Non surviors	Surviors	p Value
Ventilator days	2.56	0.59	0.001
APACHE II SCORE	27.06	13.73	0.001
SOFA SCORE	10.94	8.12	0.001
Neutropenia	6	8	0.001
Reintubation	4	10	0.001
ICU length of days	3.39	3.53	0.79
Renal replacement therapy	22	86	0.001
Vasopressors	36	116	0.002
Sex Male / female	20 / 16	262 / 188	0.76

The overall group mortality is 7.41% (36 patients) in our study is far less than calculated as per APACHE II is 18.6% for the infection.

212 sepsis patients are present in this study divided into different groups. The remaining patients are 274 which are grouped as no sepsis patients. These are represented in table 3.

**Table 3:** The data of the various group of sepsis patients.

Group	Age in years	ICU length of days	number of patients	Hospital length of days	Ventilator days mean	APACHE II Score	SOFA Score	Neutropenia	Mortality %
No Sepsis	52.2	3.2	274	9.58	0.58	12.23	7.72	6	4.37
Septicshock	58.94	3.73	148	8.47	0.9	18.18	9.36	8	13.33
Severe Sepsis	64.83	4.83	24	9.33	0.17	16.25	7.58	0	0
Sepsis	58	4.5	40	7	4	17.5	8.5	0	0

## Conclusions

The mortality and morbidity in our ICU is less compared with standard scoring systems APACHE II and SOFA. The infection rate in the ICU is less, but more resistant organisms are present. We need measures to decrease the resisstant organisms.
